# Approaches to improve *in vitro* survival, growth, and maturation of caprine oocytes: main results from LAMOFOPA-Brazil

**DOI:** 10.1590/1984-3143-AR2024-0059

**Published:** 2024-09-30

**Authors:** José Ricardo de Figueiredo, Ana Flávia Bezerra da Silva, Laritza Ferreira de Lima

**Affiliations:** 1 Laboratório de Manipulação de Oócitos e Folículos Pré-antrais, Faculdade de Veterinária, Universidade Estadual do Ceará, Fortaleza, CE, Brasil

**Keywords:** anethole, oocyte growth, *in vitro* follicle culture, caprine

## Abstract

This brief review delves into the topic of *in vitro* follicle culture for *in vitro* embryo production, with a particular emphasis on goat models. Specifically, we examine the main findings from LAMOFOPA-Brazil over the last 20 years, highlighting the challenges posed by oxidative stress and epigenetic changes. Our focus is on strategies to improve follicular development and oocyte maturation. Furthermore, we underscore the valuable role of the antioxidant anethole in optimizing the efficacy of *in vitro* follicle culture and improving outcomes in *in vitro* embryo production.

## Introduction

In the field of reproductive science, *in vitro* follicle culture (IVFC) shows great potential for retrieving and maturing follicles to support *in vitro* embryo production (IVEP). While there have been significant advancements, researchers continue to face challenges in optimizing culture conditions to mitigate oxidative stress and epigenetic factors that may impact the effectiveness of IVFC (Silva et al., 2023).

The aim of this brief review is to provide an overview of the regulation of folliculogenesis *in vivo*, as well as the fundamental aspects and applications of IVFC. Our primary focus, however, is to discuss the main approaches utilized by our research team in Brazil over the past two decades to enhance the efficiency of oocyte culture during *in vitro* maturation (IVM) and IVFC. Specifically, we will cover topics such as the activation of primordial follicles, the production of early secondary follicles from primordial follicles, the transition from late preantral to early antral follicle stages, the progression from early to late antral follicle stages, and the use of the antioxidant anethole during IVFC and IVM. We will also elucidate its further impact on IVEP, particularly in caprine species.

## Overview of folliculogenesis regulation *in vivo*

Folliculogenesis is a complex physiological process responsible for activating, growing, and maturing ovarian follicles, including preantral (primordial, transition, primary, and secondary) and antral follicles (tertiary and preovulatory). This process involves the regulation of various factors, including endocrine, paracrine, and autocrine interactions. Additionally, there are intricate signaling pathways (such as, PI3K/Akt/Foxo3 and Hippo) that control gene expression and determine cell fate, including survival or death, quiescence, or proliferation. Communication between oocytes and granulosa cells via the mTORC1 pathway plays a crucial role in initiating cellular processes required for follicle growth and maturation. For more information about folliculogenesis, see Figueiredo et al. (2018).

Based on this overview, comprehending the control of early folliculogenesis is essential to maximize the use of the abundant ovarian oocyte reserve in assisted reproductive technologies for both humans and other mammalian species. As a result, reproductive techniques, such as IVFC, which involves using non-grown oocytes enclosed in preantral and early antral follicles, and traditional IVEP methods (including IVM, *in vitro* fertilization, and *in vitro* embryo culture), which use fully-grown oocytes harvested from late antral follicles, are incredibly crucial.

## Basics of *in vitro* follicle culture (IVFC)

There are two ways to culture small follicles (preantral or early antral follicles) *in vitro*: in isolated form or enclosed within ovarian tissue, also known as ovarian tissue culture. Isolated follicles have been cultured in a two-dimensional (2D) system, where the follicle is placed on a surface that can be plastic or an extracellular matrix, such as collagen, or within an extracellular matrix (3D), such as alginate. Ovarian tissue culture, on the other hand, can be performed using ovarian slices or the entire ovary (reviewed by Figueiredo et al., 2018). To date, the most successful results of IVFC have been achieved in mice. Live pups have been born after fertilization of oocytes from *in vitro* cultured primordial follicles in mice (Eppig and Schroederc, 1989) even after freezing-thawing procedures (Liu et al., 2001). However, in other mammals, including goats, the results have been limited to the production of a small and variable number of embryos after the *in vitro* culture of isolated preantral follicles. For instance, the total number of embryos from caprine preantral follicles was 2 out of 80 (Saraiva et al., 2010), 1 out of 41 (Magalhães et al., 2011), and 2 out of 52 (Silva et al., 2015), while from early antral follicles, it was 6 out of 273 (Sá et al., 2020). The low yield of embryos in small ruminants from IVFC may be attributed to suboptimal culture medium conditions leading to excessive reactive oxygen species (ROS) production, oxidative stress, and epigenetic aberrations (Silva et al., 2023).

## Approaches to improve the survival, growth, and maturation of caprine oocytes

**Figure 1:**
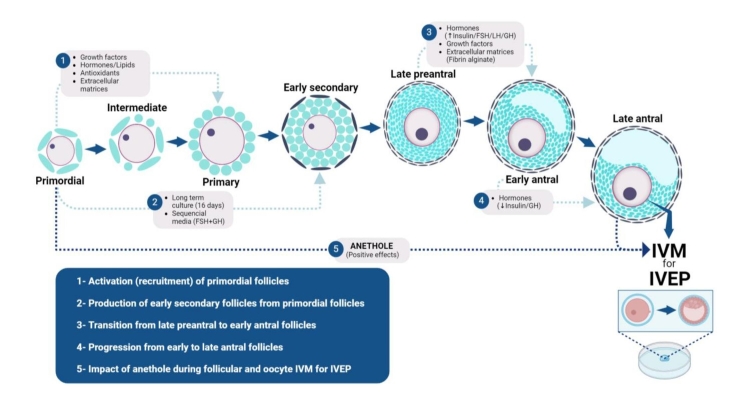
Approaches to improve *in vitro* survival, growth, and maturation of caprine oocytes. 1- Activation (recruitment) of primordial follicles; 2- Production of early secondary follicles from primordial follicles; 3- Transition from late preantral to early antral follicles; 4- Progression from early to late antral follicles; and 5- Impact of anethole during follicular (from primordial to late antral follicles) and oocyte *in vitro* maturation (IVM) for *in vitro* embryo production (IVEP). FSH: Follicle-stimulating hormone; GH: Growth hormone; and LH: Luteinizing hormone. ↑: High (10 µg/mL) and ↓: Low (10 ng/mL) insulin.

Over the past two decades, our research team in Brazil has been engaged in a significant project known as the Artificial Ovary. Within this project, the effects of more than 30 factors on the *in vitro* culture of preantral and/or early antral follicles, predominantly in caprine, have been thoroughly investigated. The primary variables examined include concentrations and combinations of factors, the type of culture system (2D *vs.* 3D, single *vs*. multiple follicle culture, short *vs.* long-term culture), fixed *vs.* dynamic culture media, among others. To provide a comprehensive understanding of the accomplishments and challenges, the IVFC process will be delineated into five sequential steps: 1) activation of primordial follicles, 2) production of early secondary follicles from primordial follicles, 3) transition from late preantral to early antral stages, and 4) growth from early antral to late antral stages and, 5) impact of anethole on follicular and oocyte culture for IVEP (Figure 1).

## Activation of primordial follicles

Follicular activation or initiation of follicle growth denotes the transition from primordial (quiescent follicle) to intermediate and primary follicle (growing follicle). This process is exceedingly complex, as evidenced by a study demonstrating that approximately 491 genes were stage-specifically up- and down-regulated during the transition from human primordial to primary follicles (Ernst et al., 2017). In our laboratory, we have investigated primordial follicle activation using ovarian tissue culture. Essentially, ovarian tissue obtained from slaughterhouse material has been cultured at 39°C in 5% CO_2_ in air in alfa-minimum essential medium (α-MEM) supplemented with 1% insulin-transferrin-selenium (ITS; 10 µg/mL insulin, 5.5 µg/mL transferrin, and 5 ng/mL selenium), 2 mM hypoxanthine, 2 mM glutamine, and 1.25 mg/mL bovine serum albumin (BSA; Correia et al., 2020). This medium served as the control medium in numerous experiments, with most IVFC experiments lasting 7 days. The key findings of the experiments in goats are summarized in Table 1. As evident from the data, the addition of various factors (including growth factors, hormones, lipids, and antioxidants) individually to the control medium increased parameters, such as follicular survival, activation, as well as follicular and oocyte diameters in a concentration-dependent manner after 7 days of culture (Figueiredo et al., 2018). Notably, most activated follicles were grouped in the growing category that included approximately 70% of intermediate and a few primary follicles (Silva et al., 2022). The results indicated that the addition of many factors individually to the culture medium improved the same endpoints. The question arises: what is a plausible explanation for this? As previously mentioned, regardless of the factor, it ultimately regulates gene expression, determining cell fate, including survival, death, quiescence, or proliferation. In this context, the Hippo signaling pathway also plays a crucial role by influencing these processes. It affects follicular growth and tissue remodeling, which can impact the effectiveness of strategies to enhance follicle activation and overall ovarian tissue culture outcomes (reviewed by Figueiredo et al., 2018). Another question that has arisen is: what is the most effective strategy to increase the percentage of activated (growing) primary follicles after 7 days of culture? We successfully enhanced primordial follicle activation to the primary follicle stage by embedding mechanically isolated primordial follicles in an alginate matrix. Utilizing this approach, 93.0% of primordial follicles developed only into primary follicles, significantly higher than other treatments, such as primordial follicles enclosed in ovarian tissue alone (70.0%) or embedded in an alginate matrix (60.0%; Correia et al., 2020).

**Table 1:** Suitable concentrations of the factors added individually during *in vitro* culture (7 days) of caprine preantral follicles enclosed in ovarian tissue: main results from LAMOFOPA.

**Parameters**	**Treatments (best concentrations) *vs.* cultured control**
↑ Survival	ANGII (10 ng/mL), BMP-15 (100 ng/mL), EGF (1 ng/mL), FGF-10 (50 ng/mL), GDF-9 (200 ng mL^-1^), IGF-1 (50 ng/mL), KL (50 ng/mL), LIF (50 ng/mL), PDGF-BB (25 ng/mL), VEGF (200 ng mL^-^1), GH (10 ng/mL), INS (10 ng/mL), VIP (10 ng/mL), S1P (1 ng/mL), AN (2000 µg/mL), AA (50 µg/mL), EUG (40 µM), JUS (0.3 mg/mL).
↑ Activation	BMP-15 (100 ng/mL), EGF (1 ng/mL), FGF-2 (50 ng/mL), GDF-9 (200 ng mL^-1^), IGF-1 (50 ng/mL), KL (50 ng/mL), LIF (50 ng/mL), E2 (10 pg/mL), GH (10 ng/mL), IAA (40 ng/mL), INS (10 ng/mL), LH (100 ng/mL), S1P (1 ng/mL), AN (30 µg/mL), EUG (40 µM).
↑ Follicular diameter	BMP-15 (100 ng/mL), FGF-2 (50 ng/mL), FGF-10 (50 ng/mL), IGF-1 (50 ng/mL), KL (50 ng/mL), PDGF-BB (25 ng/mL), TGF-β (10 ng/mL), VEGF (10 ng mL^-1^), ACT-A (100 ng/mL), FSH (50 ng/mL), E2 (10 pg/mL), GH (10 ng/mL), IAA (10 ng/mL), INS (10 ng/mL), LH (1 ng/mL), P4 (1 ng/mL), VIP (10 ng/mL), AN (30 µg/mL), AA (50 µg/mL), EUG (40 µM), MEL (1000 pM).
↑ Oocyte diameter	BMP-15 (100 ng/mL), GDF-9 (200 ng mL^-1^), IGF-1 (50 ng/mL), NGF (1 ng/mL), PDGF-BB (25 ng/mL), VEGF (10 ng mL^-1^), ACT-A (100 ng/mL), GH (10 ng/mL), IAA (10 ng/mL), VIP (10 ng/mL), AN (30 µg/mL), AA (50 µg/mL), EUG (40 µM), MEL (1000 pM).
↑ Antioxidant capacity or ↓ ROS	AN (30 µg/mL), EUG (40 µM).

Growth factors – ANGII: Angiotensin II; BMP-15: Bone morphogenetic protein 15; EGF: Epidermal growth factor; FGF-2: Fibroblast growth factor 2; FGF-10: Fibroblast growth factor 10; GDF-9: Growth differentiation factor 9; IGF-1: Insulin-like growth factor 1; KL: Kit ligand; LIF: Leukemia inhibitory factor; PDGF-BB: Platelet-derived growth factor-BB; TGF-β: Transforming growth factor beta; VEGF: Vascular endothelial growth factor. Hormones – ACT-A: Activin-A; E2: Estradiol; FSH: Follicle-stimulating hormone; GH: Growth hormone; IAA: Indole-3-acetic acid; INS: Insulin; LH: Luteinizing hormone; P4: Progesterone; VIP: Vasoactive intestinal peptide. Lipid – S1P: Sphingosine-1-phosphate. Antioxidants – AN: Anethole; AA: Ascorbic acid; EUG: Eugenol; JUS: *Justicia insularis*; MEL: Melatonin. ↑: Increased; ↓: Decreased; ROS: Reactive oxygen species.

## Production of early secondary follicles from primordial follicles

The transition from primordial to secondary follicles involves intricate coordination between oocyte-specific factors, hormonal regulation, as well as mutual signaling between oocytes and granulosa cells (Figueiredo et al., 2018). As mentioned earlier, following a 7-day culture period, primordial follicles were capable of being activated and growing into intermediate and primary follicles, yet only a few reached the secondary follicle stage. The question arises: how can the percentage of secondary follicles derived from primordial follicles be increased? A successful approach employed by our research team was to extend the culture period from 7 to 16 days and utilize sequential culture media. Essentially, we created an experimental design to investigate the additional effect of two factors, “A” and “B,” added individually or in combination during the first and/or second half of the 16-day culture period, resulting in ten treatments being tested simultaneously using follicles from the same ovarian pair. For example, in three different experiments, we examined the effects of follicle-stimulating hormone (FSH) and growth hormone (GH) (Magalhães-Padilha et al., 2012); FSH and growth differentiation factor 9 (GDF-9; Alves et al., 2013) and FSH and kit ligand/stem cell factor (KL/SCF; Lima et al., 2012). The most effective sequential media developed thus far involved the addition of FSH during the first half of the culture, followed by GH during the second half of the culture (Magalhães-Padilha et al., 2012). This treatment increased follicular survival, activation, secondary follicle formation, as well as follicular and oocyte diameters compared to the other tested treatments. However, no early antral follicles formation was observed after 16 days of culture.

## Transition from preantral to early antral follicle stage

The transition from late preantral to early antral follicles marks a critical phase in follicular growth, characterized by cavitation, where a cavity forms within granulosa cells, initiating antrum formation. This process is primarily governed by autocrine/paracrine mechanisms rather than pituitary hormones like FSH (Ferreira et al., 2018). Furthermore, this transition is accompanied by notable changes in gene expression patterns, particularly in pathways related to lipid metabolism, cell death, and the hematological system. The complexity of the transition from late preantral to early antral follicles is underscored by a study conducted by our group. This study revealed that, despite the small difference of only 150 µm in diameter between late secondary and early tertiary follicles, approximately 2,500 genes exhibited stage-specific up- and down-regulation during this transition from secondary to early tertiary follicles (Magalhães-Padilha et al., 2013). To investigate the transition from preantral to early antral follicles, late secondary follicles were mechanically isolated from the ovary using microdissection procedures and cultured individually for 18 days in 100 µL drops of a culture medium consisting of α-MEM supplemented with ITS (containing high concentration of insulin; 10 µg/mL) and recombinant bovine FSH. This medium served as the control medium in many experiments. Notably, our research group was the first to produce embryos *in vitro* following the *in vitro* culture of isolated late caprine preantral follicles using a 2D culture system. Despite the low and variable embryo production, this was achieved using a control medium supplemented with luteinizing hormone (LH) epidermal growth factor (EGF) from day 12 onwards (Saraiva et al., 2010), or GH (Magalhães et al., 2011) and vascular endothelial growth factor (VEGF; Silva et al., 2015) throughout the entire culture period. In our research, various culture systems for isolated late secondary follicles were explored to promote IVM. An important approach to enhance the efficiency of IVFC is the utilization of extracellular matrices. Comparing 2D and 3D culture systems using different matrices, fibrin alginate exhibited superior outcomes in terms of oocyte growth, meiotic resumption, and attainment of metaphase II (MII) stage (Brito et al., 2016).

## Growth of isolated early antral to late antral follicle stages

Previously reported important differences in gene expression patterns between late secondary and early tertiary follicles in goats (Magalhães-Padilha et al., 2013) suggest that the follicular requirements *in vitro* may vary between these two follicular categories. This notion was indeed validated by *in vitro* culture studies conducted by our group, which demonstrated that preantral and early antral follicles exhibited distinct behaviors in culture. For instance, under the same culture conditions using a base medium containing a low insulin concentration (10 ng/mL), the addition of human recombinant FSH improved the growth rate in early antral follicles, but not in late secondary follicles (Ferreira et al., 2018). Similarly, in another experiment, we investigated the additional effect of GH and VEGF alone or in combination on the *in vitro* growth of isolated late secondary and early antral follicles in a culture medium containing low insulin concentration (10 ng/mL). The findings revealed that the addition of GH increased oocyte diameter, percentages of fully grown oocytes, and MII rates only in early antral follicle category. This medium with low insulin (10 ng/mL) and GH was termed the early antral follicle medium and was used as a control in subsequent experiments (Cadenas et al., 2017) to evaluate the efficiency of anethole, as reported in the next section.

## Impact of anethole during follicular and oocyte culture for *in vitro* embryo production (IVEP)

Our research has achieved significant progress by incorporating anethole into the culture medium of caprine follicles and *cumulus*-oocyte complexes (COC) to regulate oxidative stress, epigenetic changes, and IVEP. Anethole, a natural phenolic substance found in essential oils of plants like *Croton zehntneri*, has demonstrated substantial antioxidant activity (Conceição-Santos et al., 2024).

In studies involving the *in vitro* culture of caprine preantral follicles enclosed in ovarian tissue, anethole increased the survival, activation, and growth of oocytes and follicles, while reducing exogenous ROS levels (Sá et al., 2018). Anethole also induced antrum formation and reduced exogenous ROS levels after *in vitro* culture of isolated late preantral follicles (Sá et al., 2017). Similarly, after *in vitro* culture of isolated early antral follicles, anethole maintained survival, increased oocyte, and follicle diameters, improved steroid secretion, reduced the expression of the pro-apoptotic gene *BAX*, and decreased exogenous ROS levels. These findings translated into higher maturation rates (57.7%) and embryonic development (10.0%) *in vitro*, leading to pregnancy following transfer of cleaved embryos (Sá et al., 2020).

In another study aimed at ensuring comparable oocyte maturation rates from both isolated follicular categories (preantral and early antral), anethole was supplemented in the basic culture medium alongside appropriate insulin concentrations and extended culture periods (24 days for preantral follicles *vs.* 18 days for early antral follicles). Interestingly, *in vitro*–derived early antral follicles exhibited comparable oocyte maturation rates to *in vivo*–derived early antral follicles (Ferreira et al., 2022).

Recent observations on IVM of caprine COC revealed that a lower concentration of anethole (30 μg/mL) increased maturation rates (73.8%) and oocyte cleavage, promoting greater viability and embryo production (47.9%) *in vitro* (Conceição-Santos et al., 2024). Furthermore, anethole increased the H3K4me3 methylation pattern, a key marker in the transcription of essential factors in oocytes and granulosa cells of early antral follicles grown *in vitro*, indicating its potential beneficial effect on epigenetic changes during the *in vitro* transition from preantral to early antral follicles (Silva et al., 2023).

## Final considerations

Advancements in IVFC and oocyte IVM, particularly in small ruminants, hold significant promise for IVEP. The incorporation of antioxidants such as anethole has demonstrated potential in enhancing IVFC efficacy and improving IVEP outcomes. Continued research is essential to refine protocols and address challenges, thus propelling assisted reproductive technologies and fertility preservation strategies forward. Regarding prospects, we have been testing not only anethole but also other compounds, including eugenol, carvacrol, and mangiferin. These compounds are being evaluated for their effects on *in vitro* culture of ovarian tissue, isolated follicles, and oocyte IVM. This approach is crucial for exploring various strategies to enhance the overall efficiency of IVFC, with the goal of producing live offspring after *in vitro* culture of goat preantral follicles.
